# Analysis of copper, zinc, arsenic, and lead content of over-the-counter toothpastes from india: an invitro study

**DOI:** 10.1038/s41598-025-12748-3

**Published:** 2025-07-22

**Authors:** S. Kavery Chengappa, Ashwini Rao, Sowmya R. Holla, Ramya Shenoy, B. H. Mithun Pai, Praveen Jodalli, B. R. Avinash

**Affiliations:** 1 Department of Public Health Dentistry Manipal College of Dental Sciences Mangalore, Manipal Academy of Higher Education, Manipal, 576 104 Karnataka India; 2 Department of Chemistry, Manipal Institute of Technology, Manipal Manipal Academy of Higher Education, Manipal, 576 104 Karnataka India

**Keywords:** Environmental sciences, Risk factors, Dentistry, Health policy, Public health

## Abstract

The regular use of toothpastes containing metals has been shown to be a formidable threat because of their ability to bioaccumulate and reach toxic proportions, affecting people’s health and the environment. While studies have evaluated the presence of metals in toothpaste across different countries, a paucity of studies in India fostered a need to identify the presence of the metals arsenic, lead, copper and zinc in over-the-counter toothpastes. The 20 most sold toothpastes were selected from Indian e-commerce platforms and prepared following quality control measures. The samples were then subjected to flame atomic absorption spectroscopy (AAS) to determine the presence and concentrations of these metals. The individual toothpaste samples presented the highest concentrations of 0.5371 mg/L arsenic, 0.0620 mg/L copper, 0.0544 mg/L lead and 7.4224 mg/L zinc. Although the concentrations of lead, copper and zinc were found to be below the permissible limits in all the toothpaste samples, the arsenic concentration of one sample exceeded the European Union (EU) standard. Arsenic, which has the potential to cause neural and gastrointestinal disorders, needs to be strictly monitored in toothpaste samples. Considering these findings, there is a need for consistent global guidelines on permissible amounts of metals in toothpastes, with a determined intention toward their implementation.

## Introduction

Toothpastes are dentifrices that are formulated with multiple acceptable components for various functions and includes binders, humectants, surfactants, abrasives/polishing agents, and other compounds that are suitable for maintaining oral health. They are a crucial requirement for maintaining optimal oral health due to their anti-bacterial, whitening, breath freshening and desensitizing functions. Their composition is carefully designed and tested for stability, optimal pH while keeping a check on toxicity of the components. Use of suitable components in their formulation have become a subject of ever evolving research and evolution with the intent of achieving better outcomes.

Toothpastes are subjected to regulations as personal care and cosmetic products or as drugs, differing based on regulations of the manufacturing country. In USA, toothpastes are regulated by the Food and Drug Administration (FDA) as over-the-counter drug if it contains fluoride^1^. Under the European Union, toothpastes are regulated as cosmetic products^2^ and in India based on whether it possesses therapeutic agents or not it is classified as drug and cosmetic respectively^3^. Although toothpastes can serve as cosmetics or drugs with therapeutic action, their regular usage as chemical products making contact with the oral mucosa more than once a day can be a matter of concern due to their constituents.

The unwarranted presence of certain elements such as metals in toothpastes has emerged as a bane to mankind and the environment owing to their long-term exposure. These metallic elements with atomic weights between 63.5 and 200.6 and specific gravities greater than 5.0 are referred to as heavy metals^4^. However, the usage of the term “heavy metals” has been disputed, with varying explanations of the term. The International Union of Pure and Applied Chemistry (IUPAC) totally challenges the usage of the term and suggests lack of scientific evidence ascribing toxicity to their mere presence^5^. The major factors that are attributed to the toxicity caused by metals among humans and environment are the duration, path of exposure and dose of the metal.

In recent years, poisoning of the environment by these metals has become a serious ecological and public health issue. Additionally, because of their exponential growth in various fields, such as industry, agriculture, households and technology, human exposure has increased considerably. Metals from personal care and cosmetic products such as toothpastes enter the environment through wastewater systems in the residual of toothpastes that are washed off. Due to lack of systems to eliminate metals from wastewater treatment plants, they enter the water bodies. Through aquatic animals and groundwater, they bioaccumulate leading to potentially entering the food chain and subsequently the ecosystem^6^.

Metals such as copper, zinc, chromium, cobalt, iron and nickel are classified as essential metals and are needed by the human body in small quantities, within specific limits. However, they have been proven to have adverse effects on human health when in excess. Arsenic, lead, cadmium, mercury and silver are classified as toxic metals and are known to be toxic to humans, causing anaemia, colic, neuropathy, sterility, gangrene and sometimes even cancer^7^. Ample evidence indicates that these metals have the potential to induce genetic damage, alter proteins and lipids and generate unbound free radicals, which can result in profound adverse effects on both human and environmental health^8^. At elevated concentrations, arsenic has been implicated in severe gastrointestinal disorders, derangements in blood composition and circulation and certain types of neural impairment^9^. Further a dose-response relationship has been established with an elevated risk of developing oral cancer associated with increased amount of arsenic ingestion^10^.

The metal copper, though beneficial in regulated amounts, can result in severe tissue damage and free radical-induced oxidative damage at excessive concentrations^11^. Lead has been shown to impact the functioning of the brain and to induce disorders of the central nervous system^12^. Although zinc is known to possess some nutritional value, this metal is toxic to humans at high concentrations. Excess ingestion of zinc can cause gastrointestinal distress and neuronal defects. Zinc toxicity has also been implicated in prostate cancer^13^.

The risk of toothpaste ingestion has been shown to be high among younger age groups and those with cognitive impairment^14^. Besides, high permeability of the oral mucosal tissue especially the sublingual and buccal mucosa facilitates the subsequent absorption of metals following chronic exposure, thus elevating the risk of metal toxicity by toothpaste use^15^. Studies have also demonstrated that chronic exposure to these metals can lead to their increased bioavailability aggravating their risk to organ systems^16^. Their constant presence in personal care products^17^ and toothpastes^18^ has been proven often with newer studies indicating metal contaminants being detected above the permissible levels. Furthermore, the consistent evidence suggesting the hazardous impact of metals on health has been studied and proven beyond doubt^19–21^. The persistence and fate of personal care products in the environment including toothpastes, further aggravate the risk caused by the metal contaminants^22^.

The increasing evidence substantiating the hazardous effects of metals on both the environment and human health has led various international organisations to prescribe limits for these potentially dangerous metals in personal care and cosmetic products, including toothpastes. The European Union Scientific Committee on Consumer Safety has recommended that the permissible limit for arsenic and lead in toothpaste should not exceed 0.1 mg/L and 0.5 mg/L respectively^2^. The United States Food and Drug Administration has suggested that the permissible limits for arsenic and lead should not exceed 5 mg/L and that other metals should not exceed 20 mg/L in personal care and cosmetic products^1^, the WHO has set limits of 10 mg/L for lead^23^. The Canadian government has set limits of 5 mg/L for arsenic and 20 mg/L for lead^24^. In India, as per the Bureau of Indian Standards (BIS), toothpastes need to comply with BIS: 6356:2001 specifications, according to which the limit of arsenic should not exceed 2 mg/L and that of other metals should not exceed 20 mg/L^3^. The Drugs and Cosmetics Rules, 1945 also specifies the prohibition of metals being added to cosmetics and mandates compliance with BIS regulations^25^. It is hence clear that the regulations in India, specific to toothpastes designate the safe limits of metal content while not completely prescribing their elimination. This contrasts with the EU regulations which specify the complete exclusion of metals in cosmetic products and only permit traces, while the US FDA and the Canadian regulations also allow for a safe limit. Having varying regulations across nations could be owing to the varying determining factors such as risk assessment, mode of usage and the regional conditions influencing the manufacturing process. Despite these regulations, different metals have been detected in toothpastes across various countries, such as Nigeria, Saudi Arabia, Malta, and Bangladesh^26–29^. Numerous studies have shown that some of the most commonly detected metals in toothpastes were lead, arsenic, copper and zinc^26–29^. The toxicity posed by exposure to lead and arsenic have been proven in various studies, while zinc and copper though essential metals can become toxic in excess concentrations^11,13^. A recent systematic review assessing the heavy metal content of toothpastes^30^ found a paucity of studies from India, which led to this study being envisaged. Further, several studies evaluating the metal content of toothpastes have either completely failed to assess compliance with international regulations or have done partial assessment with few regional regulations. The lack of clarity in literature on the brands, composition of the toothpastes in which metals were detected needs further analysis. Hence, the purpose of this study was to identify the presence of, and estimate the concentrations of arsenic, lead, copper and zinc in over-the-counter toothpastes from India and to further assess their compliance with WHO, EU, FDA and BIS regulations for toothpastes. The null hypothesis (H0) proposed for this study was that no arsenic, lead, copper and zinc would be present in these toothpastes whereas the alternate hypothesis (H1) was that evidence for the presence of arsenic, lead, copper and zinc would be obtained from the toothpastes from India.

## Methodology

The study was conducted on the most popular toothpastes in the Indian e-commerce market based on customer reviews, ratings and online visibility. The top 3 E-commerce websites with the maximum monthly visitors; Amazon, Flipkart and Myntra^31^ were assessed to select the 20 most popular toothpastes sold in India based on customer reviews, ratings and online visibility. These included different categories such as regular adult toothpastes, whitening, herbal and kids toothpastes (Table 1). All toothpaste samples were stored away from direct sunlight under dry conditions and were utilized for the study within a week of purchase. The prepared samples were subjected to flame Atomic Absorption Spectroscopy (AAS) to determine the presence and concentration of two toxic metals, arsenic and lead, and two essential metals, copper and zinc.

**Table 1:** List of toothpaste samples with their brand names and composition.

Sample number	Brand name	Ingredients
Toothpaste 1	Himalaya Sparkling white	Sorbitol, Aqua, Hydrated Silica, Glycerin, Silica, Sodium Lauryl Sulfate, Bromelain, Xanthan Gum, Titanium Dioxide, Flavor, Sodium Saccharin, Sodium Benzoate, Potassium Sorbate, Papain, Menthol, Salvadora Persica Stem Extract, Sodium Citrate, Prunus Amygdalus Dulcis Shell Extract, Cinnamomum Zeylanicum Bark Oil, Eugenia Caryophyllus Bud Oil, Thymol, Citric Acid
Toothpaste 2	Pepsodent 2 in 1	Water, Sorbitol, Calcium Carbonate, Hydrated Silica, Sodium Lauryl Sulphate, Flavor, PEG-32, Sodium Monofluorophosphate, Cellulose Gum, Trisodium Phosphate, Benzyl Alcohol, Sodium Saccharin, CI 77,891, CI 74,160, Limonene.
Toothpaste 3	Close up triple fresh formula	Sorbitol, water, hydrated silica, sodium lauryl sulphate, flavour, cellulose gum, cocamidopropyl betaine, sodium saccharin, sodium fluoride, zinc sulphate, sodium hydroxide, CI 77,891, CI 16,255, Synthetic fluorphlogopite, CI 16,035, Melaleuca alternifolia (tea tree extract), Eucalyptus globulus (eucalyptus extract), Eugenol
Toothpaste 4	Dento Shine gel toothpaste for kids	Sorbitol, Silica, DM Water, Glycerin, PEG-1500, Sodium Lauryl Sulfate, SCMC, Sodium Saccharin, Sodium Fluoride, Flavor, Sodium Benzoate, Blue CI- 42,090,
Toothpaste 5	Clove cavity protection	Calcium Carbonate, Water, Glycerin, Extracts of Babul, Bakul, Lodhra, Jamun and Akarkara, Silica, Sodium Lauryl Sulphate, Flavor containing Mint and Clove Oil, Carrageenan, Sodium Silicate, Sodium Saccharin, Sodium Benzoate, Benzyl Alcohol
Toothpaste 6	Colgate Cibaca: Anticavity toothpaste	Calcium Carbonate, Sodium Lauryl Sulphate, Sodium Silicate, Sodium Carboxymethyl Cellulose, Sodium Bicarbonate, Sodium Saccharin, Sorbitol, Silica, Flavour, Sodium Monofluorophosphate, Benzyl Alcohol, CI 42,090, In Aqueous Base.
Toothpaste 7	Colgate strong teeth	Calcium Carbonate, Water, Sorbitol, Sodium Lauryl Sulphate, Arginine, Hydrated Silica, Tatanium Dioxide, Sodium Slicate, Flavor, Carragenan, Sodium Monofluorophosphate, Potassium Nitrate, Sodium Bicarbonate, Benzyl Alcohol, Sodium Saccharin, Limonene.
Toothpaste 8	Lever Ayush: freshness gel cardamom toothpaste	Elletaria cardamomum, Mentha pipera, Arimedas tailam, Ashtang hridayam, CI 42,090, CI 47,005, Sodium fluoride
Toothpaste 9	Colgate Herbal toothpaste	Calcium Carbonate, Aqua, Sorbitol, Sodium Lauryl Sulfate, Hydrated Silica, Aroma, Sodium Monofluorophosphate, Cellulose Gum, Magnesium Aluminum Silicate, Sodium Saccharin, Sodium Carbonate, Benzyl Alcohol, Sodium Bicarbonate, Ananas Sativus Fruit Juice, Citrus Aurantium Dulcis Juice, Citrus Limon Juice, Fragaria Ananassa Fruit Juice, Mangifera Indica Juice, Camellia Sinensis Leaf Extract, Anthemis Nobilis Flower Oil, Commiphora Myrrha Oil, Eucalyptol, Salvia Officinalis Oil, Limonene, CI 74,260, Sodium Monofluorophosphate
Toothpaste 10	Colgate Sensitive toothpaste	Potassium Nitrate, Sodium Fluoride, Water, Glycerin, Hydrated Silica, Sorbitol, PEG-12, Tetrapotassium Pyrophosphate, PVM/MA Copolymer, Flavor, Sodium Lauryl Sulfate, Poloxamer 407, Sodium Hydroxide, Sodium Saccharin, Cellulose Gum, Xanthan Gum, Titanium Dioxide
Toothpaste 11	Colgate Active Salt toothpaste	Calcium Carbonate, Sorbitol, Silica, Sodium Lauryl Sulfate, Sodium Silicate, Sodium Monofluorophosphate, Sodium Carboxymethyl Cellulose, Sodium Chloride, Sodium Bicarbonate, Sodium Saccharin, Xanthan Gum, Benzyl Alcohol, Pigment Blue 15/​Ci 74,160
Toothpaste 12	K.P. Namboodiri’s Natural Salt toothpaste	Calcium carbonate, Rock salt, Sodium bicarbonate, Eugenia caryophyllus (Clove) extract, Zingiber officinale (Ginger) root extract, Melaleuca alternifolia (Tea tree) leaf Oil, Myristica fragrans (Nutmeg) extract, Elettaria cardamomum (Cardamom) extract, Menthol, Mentha piperita (Peppermint) oil
Toothpaste 13	Dabur Meswak	Calcium Carbonate, Sorbitol, Water, Silica, Sodium Lauryl Sulphate, Flavour, Miswak Extract, Cellulose Gum, Carrageenan, Sodium Silicate, PVM/MA Copolymer, Sodium Saccharin, Zinc Gluconate, Sodium Benzoate
Toothpaste 14	Colgate Max fresh	Sodium Fluoride, Sorbitol, Water, Hydrated Silica, PEG-12, Sodium Lauryl Sulfate, Flavor, Cellulose Gum, Tetrasodium Pyrophosphate, Cocamidopropyl Betaine, Sodium Saccharin, Methylcellulose, Titanium Dioxide, FD&C Blue No.1.
Toothpaste 15	Sensodyne fresh mint	Potassium nitrate, Sodium fluoride, Water, sorbitol, hydrated silica, glycerin, cocamidopropyl betaine, flavor, xanthan gum, titanium dioxide, sodium saccharin, sodium hydroxide, sucralose, yellow 10, blue 1
Toothpaste 16	Neem active toothpaste	Precipitated Calcium Carbonate, Sorbitol, Aqua, Glycerine, Hydrated Silica, Sodium Lauryl Sulphate, Flavour, Sodium Silicate, Sodium Carboxymethylcellulose, Carrageenan, Sodium Saccharin, Methylparaben, Propylparaben, Neem Extract, Sodium Monofluoro Phosphate, Sodium Dihydrogen
Toothpaste 17	Vicco vajradanti: saunf flavour	Ajwain Trachyspermum, Akarkara, Anant Mool, Barleria Prionitis, Bor (Zizyphus Jujuba), Caryophyllus Aromaticus Fl, Dalchini, Glycyrrhiza Glabra Extract, Gum Acacia (acacia Arabica), Jamun, Juglans Regia (Walnut Oil), Kabab Chini, Khadira (Acacia Catechu), Manjistha, Mimusopa Elengi, Patranga, Quercus Infectoria Galls, Triphala
Toothpaste 18	Colgate Charcoal clean	Sorbitol, Water, Silica, Sodium Lauryl Sulphate, Flavour, Cocamidopropyl Betaine, Polyethylene Glycol 600, Sodium Carboxymethyl Cellulose, Sodium Saccharin, Sodium Fluoride, Charcoal, Benzyl Alcohol, Eugenol
Toothpaste 19	Colgate Total advanced health	Glycerin, Silica, Sodium Lauryl Sulphate, Arginine, Flavor, Cocoamidopropyl Betaine, Zinc Oxide, Sodium Carboxymethyl Cellulose, Titanium Dioxide, Poloxamer 407, Zinc Citrate Trihydrate, Tetrasodium Pyrophosphate, Xanthan Gum, Benzyl Alcohol, Phosphoric Acid, Sodium Saccharin, Sodium Fluoride, Titanium Dioxide Coated Mica, Sucralose, CI 74,260, CI 47005:1, Eugenol, in aqueous base
Toothpaste 20	Close up ever fresh: lemonmint	Sorbitol, water, hydrated silica, sodium lauryl sulphate, PEG-32, Flavor, cellulose gum, sodium saccharin, sodium fluoride, zinc sulphate, sodium hydroxide, CI 77,891, Synthetic Fluorphlogopite, CI 47,005, CI 42,090, Citral, Limonene

### Quality control measures

Reagent blanks for deionized water and nitric acid were made and analysed along with the samples to check for contamination. For calibration of the Atomic Absorption Spectrometer a series of standard solutions were prepared from certified standard stock solutions. All the glassware used were soaked in 10% nitric acid overnight and thoroughly rinsed with deionized water before use to prevent contamination.

### Preparation of the toothpaste samples for atomic absorption spectroscopy (AAS)

Blinding was performed by masking and numbering the toothpastes. The samples were prepared by mixing 1.0 g of each toothpaste sample with 50 mL of deionised water in a 100 mL beaker. The solution was digested with 5 mL of concentrated nitric acid. This procedure was carried out on a hotplate at a temperature of 140 °C for 2 h under atmospheric pressure, until a clear mixture was obtained, suggesting complete digestion of the samples. The mixture was allowed to cool and then centrifuged in a centrifuge of rotor radius 10 cm for 5 min at 1368 g-force at room temperature. The supernatant was then transferred to a 100 mL volumetric flask. The solution was then made up to the required volume of 100 ml using deionised water^29^.

### Atomic absorption spectroscopy (AAS)

The prepared samples were subjected to flame atomic absorption spectroscopy (AAS) to determine the metal content. A Thermo Scientific™ iCE™ 3000 Series AAS instrument was used for this study. The prepared samples of each toothpaste along with the standard solution for each metal were subjected to flame AAS, and the concentrations of the specific metals in the prepared toothpaste samples were determined.

AAS detects elements in liquid or solid samples by applying characteristic electromagnetic radiation of a specific wavelength from a light source. Flame AAS utilises sample atomisation when a liquid sample is drawn into a flame. The flame performs the role of a “sample holder,” following the passing of the light through the atoms and flame simultaneously, thus determining the absorbance of light. With an appropriate wavelength, the absorption of light can be determined with the help of a standard curve^32^.

Since the prepared toothpastes were checked for the presence of arsenic, lead, copper and zinc, the standard solutions were made of these metals at different concentrations. The calibration curve for arsenic was set with standard solutions having concentrations of 10 mg/L, 20 mg/L, 30 mg/L, 40 mg/L and 50 mg/L. The calibration curve for Arsenic was a linear segmented curve having characteristic concentration of 0.8011 mg/L. For lead, the calibration curve was constructed with standard solutions of 1 mg/L, 3 mg/L, 5 mg/L, 7 mg/L, and 10 mg/L. The calibration curve showed linear relation with the concentrations of the standard solutions. The characteristic concentration was 0.1862 mg/L. For copper, the calibration curve was constructed with standard solutions of concentrations 1 mg/L, 2 mg/L, 3 mg/L, 4 mg/L, and 5 mg/L, and the curve showed a linear relation with the concentrations. The characteristic concentration obtained was 0.0764 mg/L. For zinc, the calibration curve was established with standard solutions of 0.5 mg/L, 1.0 mg/L, and 1.5 mg/L. The calibration curve was linear in relation to concentrations of the standard solutions and the characteristic concentration was 0.0198 mg/L. All standard solutions showed low Relative Standard Deviation (RSD) of less than 1.5% suggestive of higher precision.

## Results

Four metals, arsenic, lead, copper and zinc, were found to be present in all 20 toothpaste samples tested. However, the concentrations of these metals varied, and among the 20 samples studied, samples 1, 4 and 3 presented relatively high concentrations of arsenic taking into consideration the European Union regulations^2^, at 0.5371 mg/L, 0.2663 mg/L and 0.2424 mg/L, respectively, compared with those of the other samples. The concentration of lead was found to be highest in sample 8 at 0.0544 mg/L, and high concentrations of copper were found in samples 2, 7 and 6, at 0.0620 mg/L, 0.0467 mg/L and 0.0377 mg/L, respectively, compared with the other samples. For zinc, the highest concentrations of 7.4224 mg/L, 2.5002 mg/L and 1.8501 mg/L were observed in samples 18, 13 and 8, respectively (Table 2).

**Table 2:** Metal concentrations of the toothpaste samples obtained by AAS and compared with various regulatory frameworks.

TYPE	BRAND NAME	SAMPLE NO.	COPPER	ZINC	ARSENIC	LEAD	COMPARISON WITH REGULATORY LIMITS
Whitening		Toothpaste 1	0.016	0.060	0.54	0.029	Arsenic exceeds EU regulations
		Toothpaste 3	0.009	0.98	0.24	0.035	Arsenic exceeds EU regulations
Herbal/ayurvedic/mint/lemon mint/		Toothpaste 5	0.034	0.055	0.14	Below LOD	Compliant
		Toothpaste 8	0.007	1.85	0.095	0.054	Compliant
		Toothpaste 9	0.031	0.055	0.032	0.004	Compliant
		Toothpaste 13	0.034	2.50	0.16	Below LOD	Compliant
		Toothpaste 15	0.005	0.018	0.015	Below LOD	Compliant
		Toothpaste 16	0.029	0.069	Below LOD	0.024	Compliant
		Toothpaste 17	0.0036	0.023	0.11	0.040	Compliant
		Toothpaste 20	0.027	0.11	0.028	0.009	Compliant
Anti- bacterial/germ fighting		Toothpaste 2	0.062	0.042	0.18	Below LOD	Compliant
		Toothpaste 19	0.0032	2.30	0.10	0.023	Compliant
Kids		Toothpaste 4	0.006	0.034	0.27	0.030	Arsenic exceeds EU regulations
Anti-cavity		Toothpaste 6	0.038	0.045	0.16	Below LOD	Compliant
Salt (active salt/natural salt)		Toothpaste 11	0.029	0.095	0.053	0.001	Compliant
		Toothpaste 12	0.029	0.057	0.081	0.003	Compliant
Everyday Protection		Toothpaste 10	0.0056	0.032	0.16	0.019	Compliant
Calcium		Toothpaste 7	0.047	0.048	0.11	Below LOD	Compliant
Cooling		Toothpaste 14	0.004	0.041	0.036	0.042	Compliant
Charcoal		Toothpaste 18	0.002	7.42	0.068	0.039	Compliant

The readings obtained below the instrument’s Limit of Detection (LOD) are noted as “below LOD.” All readings are rounded off for analytical precision. Limit of Detection (LOD) for copper = 0.002 mg/L, Zinc = 0.005 mg/L, Lead = 0.0005 mg/L and Arsenic = 0.001 mg/L.

- BIS: 6356:2001 specifications direct the limit of arsenic to not exceed 2 mg/L, other metals to not exceed 20 mg/L.
- The European Union Scientific Committee on Consumer Safety sets limits of 0.5 mg/L for lead and 0.1 mg/L for arsenic.
- World Health Organization prescribes a limit of 10 mg/L for lead.
- The Food and Drug Administration (USA) set limits of 5 mg/L for lead and 20 mg/L for other metals.

Although all the samples contained metals, the concentrations of these metals were within the permissible limits prescribed^1–3,23,24^, except for toothpaste 1, which had an arsenic concentration of 0.53 mg/L, which exceeded the European Union standard for arsenic in toothpaste. However, the international standards used for comparison, each vary based on their scientific contexts. The Arsenic limits in toothpaste 1 were compliant with other regulations, while exceeding the European Union standards. This emphasizes the need to have evaluation of toothpastes specific to the context of the regulatory standards.

Some of the toothpaste samples exhibited very low concentrations. The limits of detection (LOD) for this instrument using flame AAS are 0.05 mg/L for arsenic, 0.007 mg/L for lead, 0.003 mg/L for zinc and 0.004 mg/L for copper. The observed concentrations in some samples were noted to be below or close to these detection limits, this may affect the accuracy of quantification. Establishing limits of detection that are specific to the method or validating the methods could improve the reliability of the results, which needs to be included in future studies. Also, the lack of replicate analysis and purely descriptive nature of this study without statistical analysis implies that the results need to be analysed prudently.

## Discussion

Studies across different countries have reported the presence of metals in varying quantities in a wide range of personal care and cosmetic products^26,27^. The variation in proposed permissible limits of lead, arsenic and other metals by various regulatory bodies, is the result of focusing on different factors for proposing the limits. The FDA presents limits based on risk of exposure to these metals as color additives and intentional ingredients^1^. The WHO provides the limits as health-based guidelines for toxic metals^23^. While the regional manufacturing practices and the regulatory approach followed by each of these institutions could be the possible reason behind the difference, it is also important to consider factors specific to toothpastes as opposed to other personal care and cosmetic products. These factors include the route of exposure, risk of ingestion especially among younger children leading to stricter regulations compared to other cosmetic products^6^.

In this study, all 20 samples of over-the-counter toothpastes from India contained metals, arsenic, copper, lead and zinc. Although the concentrations of these metals were found to be very low and within the prescribed standards, their chronic uptake into the body at regular low concentrations might result in toxic reactions cumulatively, with serious consequences over time. A study conducted to analyse the metals concentration of dentin found 0.27 to 6.16 µg/g of lead, 0.17 to 1.10 µg/g of Arsenic, 0.22 to 15.7 µg/g of zinc and 0.12 to 12.7 µg/g of copper in different tooth samples across age groups. Clearly, this highlights the potential health risks of metals due to chronic exposure from various sources including toothpastes^33^.

Arsenic is known to have hazardous effects on the human body, causing melanosis, hyperkeratosis, gangrene and even known skin cancer^34^. Only one sample in the present study was found to have no arsenic, and for the remaining samples, the concentration ranged from 0.0277 to 0.5371 mg/L. Two of the three samples with arsenic concentration exceeding EU limits were whitening toothpastes and the third sample was found to be a children’s toothpaste. Considering the children’s toothpaste demonstrated to exceed the limits is concerning as studies have shown that children have a greater risk of ingestion of toothpastes^14^. The composition of both toothpaste 1 and 3 show natural extracts, their extraction without complete purification could have led to the elevated arsenic levels.

Further comparing the results of our study to other studies, it was found that slightly lower values of arsenic, in the range of 0.0006 mg/L to 0.0269 mg/L, were reported by Salama^27^ in 4 toothpastes tested in Saudi Arabia. Paul et al.^29^, reported arsenic concentrations in the range of 0.027–0.637 mg/L in toothpastes from Bangladesh. However, Almukainzi et al.^35^, in their study of 2 toothpastes from Saudi Arabia, reported very high concentrations of arsenic ranging from 221.96 to 209.33 mg/L. These vast variations across studies in the concentration of arsenic detected, could be attributed to the use of different analytical techniques such as Inductively Coupled Plasma–Mass Spectrometry (ICPMS) in some of the studies^27,35^ as compared to AAS used in others^29^. Another notable factor is the different sample preparation methods utilized in each of these studies using different reagents for sample digestion could also have resulted in this variation.

Lead poisoning can cause irritability, loss of appetite, blue‒black gum deposits, weight loss, vomiting, constipation, anaemia, and renal failure^36^. In the present study, 5 samples showed concentration of lead below the limit of detection suggesting non-detectable quantity, while in the remaining samples, the concentration of lead ranged from 0.006 to 0.0544 mg/L. In a study conducted by Odukudu et al.^37^ for a 0.02 mg/L concentration of lead in their study on toothpaste in Nigeria. However, another study^26^ from Nigeria reported very high concentrations of lead ranging from 4.514 to 23.575 mg/L in 35 samples, which was very high. It can be noted here that both these studies from Nigeria show varying concentrations of lead, one of the major reasons for this could be the absence of regulations on metal content of personal care products. Consequentially, various studies across Nigeria on personal care products including toothpastes showed high concentration of lead. Additionally, Eneh et al.^38^ found elevated levels of lead (17.3 µg/dL) in the urine samples of individuals who used personal care products as compared to non-users, raising concerns about lead contamination. High concentrations of lead have also been reported by Salama^27^ in Saudi Arabia (0.1856–0.6313), Paul et al.^29^ from Bangladesh (0.27–2.12), Almukainzi et al.^35^ from Saudi Arabia (75.86–78.31) and Lawi et al. from Iraq (1.00–12.05)^39^. These substantial variations in lead concentration across studies from different countries and toothpastes having varying formulations is likely due to dissimilarity in the manufacturing of toothpastes, differing regulations and varied methods used for their analysis.

However, in their study of 10 toothpastes from Nigeria, Osi and Ekpete^40^ reported that 8 of them did not contain copper, and the remaining two had concentrations ranging from 0.01 to 0.02 mg/L, which was similar to the present study. In the present study, the concentration of copper was found to range from 0.0022 to 0.0620 mg/L. It was also observed that copper concentration of herbal toothpastes was found to be slightly higher than other samples, the use of plant extracts rich in copper could be contributing to this variation. The copper concentrations reported by various studies included Odukudu et al.^37^ from Nigeria [0.23], Salama^27^ from Saudi Arabia (0.5590–2.2988), Vella & Attard^28^ from Malta (0.73–3.68), Paul et al.^29^ from Bangladesh (2.78–13.1), and Almukainzi et al.^35^ from Saudi Arabia (264–269.94). Osi and Ekpete^40^, in their study on the copper content of toothpastes from Nigeria, reported that although 5 out of the 10 samples did not contain copper, the concentration of copper was in the range of 1.47 to 1.83 mg/L in the remaining 5 samples, which was higher than that reported in the present study. One of the reasons for the very high concentration of copper in toothpaste samples of these studies could be due to use of oxide of copper being used as a pigment. While direct similarity cannot be implied between the results of our study with other studies, it is considerable that the lower concentrations are suggestive of different formulations, or more stringent adherence to regulatory limits in Indian toothpastes.

In the present study, all twenty samples contained zinc at concentrations ranging from 0.018 to 7.4224 mg/L. The highest concentration of zinc was observed in the charcoal-based toothpaste, suggestive of complete oversight of the regulatory norms with absence of its mention in the composition claim of the toothpaste. Osi and Ekpete^40^, reported concentrations ranging from 0.01 to 3.92 mg/L, and very low concentrations of zinc, 0.2555 mg/L, were reported by Odukodu et al.^37^ in Nigeria, comparable to the findings of this study. Another study from Nigeria (Ogidi and Agbo)^41^ evaluated five toothpastes and reported the presence of zinc in the range of 1.19–3.08 in 3 samples and very high concentrations of 84.67 and 81.27 mg/L in two samples. Vella & Attard^28^, in their study of 9 toothpastes in Malta, reported that although one toothpaste was found to be completely free of zinc, seven toothpastes contained zinc, ranging from 0.31 to 7.8 mg/L which was similar to the results of the present study. However, Vella & Attard^28^ reported that one toothpaste had a zinc concentration of 2417 mg/L, which was very high compared with that reported in the present study. The studies showing high values are indicative of potential contamination or presence of a zinc-based compound that is highly concentrated. Lawi et al.^39^ studied the zinc concentration of toothpastes in Iraq and reported that although 9 of the toothpastes contained zinc in the range of 1.59 to 11.3 mg/L, one toothpaste had a zinc concentration of 402.34 mg/L, which was remarkably high. This sample had a plant- based formulation and hence may have led to the extreme concentration due to the trace elements. With most of the results showing herbal or unconvential toothpastes having higher metal concentration, stringent regulation and transparency of ingredients is crucial. Studies have shown that herbal components used in manufacture of cosmetics inherently absorb metals and show elevated levels, which could contribute to metal contamination as seen in our study^42^. The assessment of metals being done through different techniques could be a possible explanation for the variability in the study’s results. Each of these instruments have distinct limits of detection, sensitivity and specificity, which can alter the results at the level of trace metals^43^. The commonly employed methods of Atomic Absorption Spectroscopy (AAS) and Inductively Coupled Plasma Mass Spectroscopy (ICPMS) differ in their sensitivity in metal detection, with ICPMS being more sensitive^43^. Further the ability of ICPMS to detect multiple metals at the same time and its detection limits that is lesser than other methods such as AAS enable more accurate detection^44^. These factors could influence the detection of metals in this study. Most studies employ varying digestion methods for the toothpastes, to dissolve them into solutions before being assessed, which could further be based on proper digestion, absence of contamination causing wrong estimations. The studies have considered variety of brands of toothpastes with diverse formulations being assessed. The toothpastes included those manufactured locally, natural ingredient-based formulations which have the potential of containing unregulated additives in the herb-based extracts contributing to the elevated metal levels. The presence of metals in one of India’s major rivers and its sediment bed has been evaluated and established; the water and sediments used in manufacture of toothpastes could be possible sources of metal contamination^45–49^.

Metal contamination has become a global concern due of its toxicity, environmental persistence, bioaccumulation, and biomagnification. The daily use of toothpastes containing metals can be a threat since their chronic use can cause systemic and environmental reactions affecting people’s health and the environment. Metals such as arsenic and lead are among the chemicals that are not needed by the human body and can be completely avoided^34^.

Copper is an essential trace element required by the human body in small amounts. It is required for the immunological response, neuropeptide production, antioxidant defense, and iron metabolism. Despite being a necessary micronutrient for humans, excess copper is toxic and can lead to cell death and oxidative damage^11,50^. Zinc is essential for growth and development. It plays important roles in reproduction, immunology, neuronal growth, DNA metabolism and repair, eyesight, taste, and cognition. However, excessive intake of zinc induces copper deficiency and causes changes in immunological parameters and lipid profiles^51^.

### Limitations

While this study was performed to highlight the presence of metals, arsenic, lead, copper and zinc in 20 samples of toothpastes from India, there is a need to assess the presence of other metals as well. Evaluation of all the toothpastes in the Indian market, considering the batches and manufacturing dates, which this study did not account for could aid in assessing the effective implementation of the laws to uphold public health. Further, the toothpastes were selected depending on their online popularity across only 3 e-commerce platforms indicated by customer reviews and ratings instead of sales data. This could restrict the true market representativeness of the toothpaste samples. The use of convenience sampling for selection of toothpastes results in selection bias and risks overlooking the content of toothpastes not sold online. Additionally, e-commerce platforms can algorithmically promote products of certain brand or user preferences, hampering generalizability of the results. The standard solutions used for calibration were not aligned with the low concentrations observed in the test samples. This can impact the quantification of trace metals. Replicate analysis was not done for the toothpaste samples and absence of standard deviation reporting affects the reproducibility of the results. The study could identify only the presence of arsenic, lead, copper and zinc. Therefore, statistical analyses across samples or significance testing could not be conducted. Due to convenience sampling and a small sample size, inferential statistical analysis was not carried out since generalizability would then be misleading. Also, considering the purely descriptive nature of this study, uncertainty analysis and confidence intervals were not calculated and only concentrations of metals in toothpaste samples were reported. Further, the observations made through this study are presented descriptively, thus no conclusive comparisons can be drawn.

## Conclusion

This study highlights the presence of metals in over-the-counter toothpastes from India. The study revealed that the metal concentrations of lead, copper and zinc were below the permissible limits in all the toothpaste samples. However, the arsenic concentration of one sample exceeded the EU standard. Considering the limitations of this study, without evidence on the chronic exposure and bioaccumulation of metals from toothpastes, concluding the safety of toothpastes would be unduly over estimation. The presence of metals in personal care and cosmetic products such as toothpastes has in the recent times become a major ecological and public health problem owing to their potential to cause harmful effects on human health as well as the environment. National and international regulations restricting the presence of metals in personal care products, including toothpastes, are very broad. Large scale research across brands, from different countries, with robust study designs need to be undertaken to provide scientific evidence, thus influencing the framing of consistent guidelines across the globe. Continuous monitoring of toothpastes should be adopted to ensure optimum health and quality of life of the population.

## Data Availability

All data generated or analysed during this study are included in this published article.

## Declarations

### Clinical trial registry details

Not applicable since it is not a clinical trial.
